# Association between red cell distribution width to albumin ratio and 28‑day mortality in older patients with sepsis: A retrospective cohort study

**DOI:** 10.1097/MD.0000000000045835

**Published:** 2025-11-07

**Authors:** Kai Hu, Husun Qian

**Affiliations:** a Clinical Laboratory Center, The Affiliated Hospital of Guizhou Medical University, Guiyang, Guizhou, P.R. China.

**Keywords:** 28-day mortality, albumin, older patients, red cell distribution width, sepsis

## Abstract

Red blood cell distribution width to albumin ratio (RAR) is a novel biomarker and its prognostic effect on critically ill patients with sepsis has not been extensively investigated. The objective of this study was to investigate the association between RAR and prognosis in older patients with sepsis. We performed a retrospective cohort study utilizing the eICU Collaborative Research Database to examine the relationship between the RAR and patient outcomes in sepsis cases. The primary endpoint was all-cause mortality within 28 days of intensive care unit admission. To explore this association, we employed multivariate regression analysis and conducted subgroup analyses. Additionally, receiver operating characteristic curves and Kaplan–Meier survival analysis were utilized to assess prognostic value and survival differences, respectively. The study included 17,321 eligible patients. It was observed that the median of RAR was notably higher in patients who did not survive compared to those who did at the 28-day mark. Participants were categorized into 3 groups based on their RAR values, revealing a significantly increased risk of 28-day mortality in the group with elevated RAR. The association between RAR and 28-day mortality risk appeared potentially nonlinear. Kaplan–Meier survival analysis indicated that those in the higher RAR group experienced increased 28-day mortality. Our study shows that RAR is significantly associated with poor clinical prognosis in sepsis. The higher RAR is an independent predictor of 28-day mortality in older patients with sepsis.

## 1. Introduction

Sepsis is a systemic inflammatory response syndrome caused by infection. It is common in patients with severe trauma or infectious disease.^[1,2]^ Its pathogenesis includes infection caused by bacteria, fungi, viruses and parasites, which leads to the imbalance of inflammatory reaction and immune regulation.^[3,4]^ With the increasing aging population, the incidence of sepsis in the elderly and its burden on society are continuously rising. It predominantly affects older adults due to their decreased immune function and increased comorbidities.^[5]^ Despite advancements in medical care, the mortality rate among older patients with sepsis remains high.^[6]^ Therefore, identifying reliable prognostic markers is critical for improving management and outcomes in this vulnerable population.

Currently, various clinical features and biomarkers are used for predicting mortality risk in elderly patients with sepsis. Among them, red blood cell distribution width to albumin ratio (RAR) have gained widespread attention as common inflammatory markers in recent years. Red cell distribution width (RDW) reflects the size and morphological variation of red blood cells, while the albumin reflects the nutritional status and degree of inflammation in the body.^[7,8]^ Both of these indicators can be obtained quickly through routine blood tests, providing the advantages of simplicity and low cost.

Previous studies have indicated that RAR may play a crucial role in predicting mortality rates in elderly patients with sepsis.^[9–11]^ Firstly, an elevation in RDW is commonly associated with increased inflammation and tissue damage, while a decrease in albumin ratio reflects the severity of the inflammatory state.^[12,13]^ The RAR has been proposed as a novel index that combines the prognostic capabilities of both RDW and albumin. It reflects the balance between inflammation and nutritional status, potentially offering a more comprehensive assessment of a patient’s condition. Therefore, RAR can serve as indicators reflecting systemic inflammation, helping to assess the severity of the disease in elderly patients with sepsis. RAR may also be associated with immune function and inflammatory regulation in elderly patients with sepsis. Immunosenescence is an important factor contributing to increased susceptibility to infections and progression to severe complications in elderly patients with sepsis.^[14]^ Some studies have suggested that RAR may be related to immune cell function and inflammatory pathways, thereby influencing the mortality risk in elderly patients with sepsis.^[15,16]^

However, there is still controversy regarding the role of RAR in predicting mortality rates in elderly patients with sepsis. Some studies have found a significant correlation between high RDW, low albumin ratio, and mortality rates in elderly patients with sepsis, while others have not observed such associations.^[17–20]^ Therefore, further research is needed to clarify the role and mechanisms of RAR in predicting mortality risk in elderly patients with sepsis.

This retrospective cohort study aims to investigate the association between RAR and 28-day mortality in older patients with sepsis. By exploring this relationship, we seek to enhance the understanding of RAR as a prognostic tool and contribute to better risk stratification and management strategies in this high-risk group, ultimately reducing mortality rates and improving patient quality of life.

## 2. Methods

### 2.1. Data sources

This study was a retrospective observational analysis utilizing data extracted from the eICU Collaborative Research Database (eICU-CRD), an international online resource. The eICU-CRD is a multicentre database containing detailed information on over 200,000 intensive care unit (ICU) admissions across the United States, part of the eICU program. Database access was secured by passing an examination and obtaining certification as per the data usage agreement of the PhysioNet Review Board. Due to the study’s retrospective nature, absence of direct patient interventions, and privacy measures meeting safe harbor standards as certified by Privacert (Cambridge), it was exempt from approval by the institutional review board of the Massachusetts Institute of Technology (record ID: 40859994). Consequently, informed consent was waived. The study was conducted in alignment with the Declaration of Helsinki, adhering to all pertinent guidelines and regulations.

### 2.2. Study population

**Figure 1.**
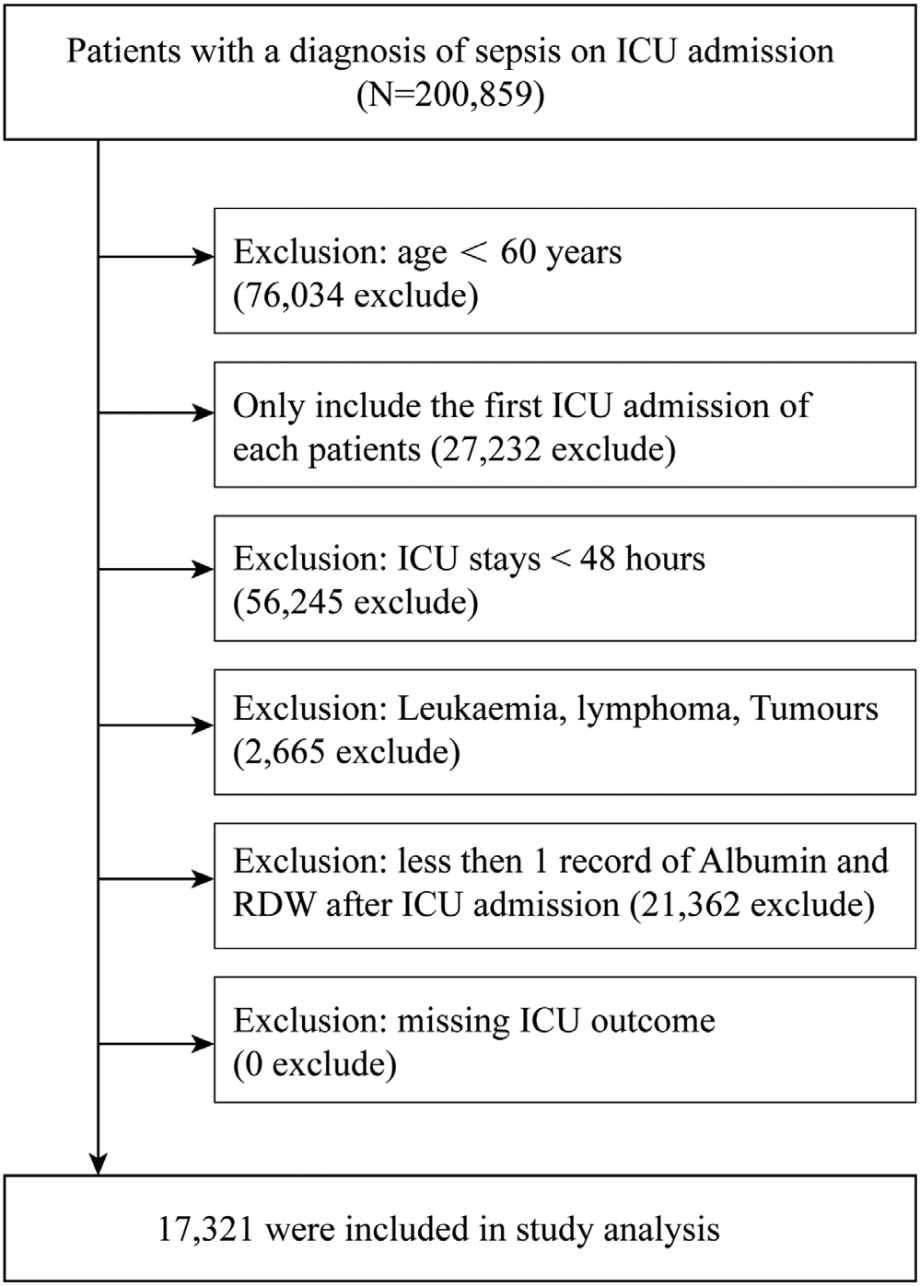
Flow chart of study population enrolled from the eICU-CRD. eICU-CRD = eICU Collaborative Research Database.

All patients identified with sepsis at the time of ICU admission were considered for inclusion. The study applied the following exclusion criteria: not the initial ICU admission, ICU stay <48 hours, age under 60 years, absence of ICU outcome data, incomplete records of total RDW and albumin post-ICU admission or data loss due to system errors, and diagnosis of leukaemia, lymphoma, or tumors. The flowchart depicting the study design is shown in Figure 1.

### 2.3. Missing data explanation

Because the missing rate for each variable in the study was <5 %, serial interpolation was not used to handle missing data.

### 2.4. Study endpoints

The outcome of the study was all-cause ICU mortality within 28 days after admission to the ICU.

### 2.5. Statistical analysis

Participants were categorized into 3 groups based on the tertiles of the red cell distribution width to albumin ratio (RAR) for descriptive statistical analysis. Continuous variables were summarized as mean ± standard deviation or median with interquartile range (IQR), and compared across groups using the *t*-test or one-way ANOVA, as appropriate. Categorical data were presented as frequencies or percentages and analyzed via the Chi-square test or Fisher exact test.

To explore the association between RAR and 28-day mortality in older septic patients, we utilized both univariate and multivariate binary logistic regression models. We reported unadjusted models (Model 1), models adjusted for demographic factors (Model 2), and fully adjusted models with all covariates listed in Table 1 (Model 3). Results were presented as odds ratio (OR) with 95% confidence intervals.

**Table 1:** Baseline characteristics of participants (N = 17321).

Characteristic	RAR			
	Tertile 1, <4.82	Tertile 2, 4.82–6.30	Tertile 3, >6.30	*P*-value
RDW-to-albumin ratio	4.13 ± 0.47	5.50 ± 0.42	8.27 ± 2.12	<.001
Number	5764 (33.28%)	5778 (33.36%)	5779 (33.36%)	–
Age (year)	72.98 ± 8.60	74.29 ± 8.77	73.76 ± 8.68	<.001
Gender				
Male	2536 (44.0%)	2750 (47.6%)	2866 (49.6%)	<.001
Female	3228 (56.0%)	3028 (52.4%)	2913 (50.4%)	
BMI	28.94 ± 7.42	29.22 ± 8.41	28.36 ± 8.34	<.001
WBC	10.60 (7.80–15.20)	11.00 (7.50–16.56)	12.90 (7.90–19.00)	<.001
Temperature	36.36 ± 1.06	36.36 ± 1.13	36.28 ± 1.18	<.001
Heart rate	98.08 ± 32.24	103.02 ± 31.31	107.68 ± 30.36	<.001
Acute physiology score III	47.92 ± 23.12	55.00 ± 24.28	63.92 ± 25.30	<.001
Apache IV score	63.82 ± 23.55	71.77 ± 24.59	80.77 ± 25.51	<.001
GCS score	12.29 ± 3.80	12.10 ± 3.81	11.92 ± 3.79	<.001
AST	30.00 (20.00–61.00)	32.00 (20.00–68.00)	33.00 (20.00–69.00)	<.001
ALT	25.00 (16.00–42.00)	26.00 (16.00–48.00)	25.00 (15.00–48.00)	.001
Total protein	6.33 ± 0.77	5.83 ± 0.81	5.38 ± 0.96	<.001
Albumin	3.45 ± 0.40	2.83 ± 0.34	2.17 ± 0.44	<.001
Lactate	1.90 (1.20–3.02)	1.80 (1.10–3.00)	1.90 (1.20–3.20)	.125
RBC	3.93 ± 0.71	3.59 ± 0.73	3.34 ± 0.72	<.001
RDW	14.13 ± 1.17	15.50 ± 1.71	17.35 ± 2.82	<.001
COPD				
No	5164 (89.59%)	5084 (87.99%)	5191 (89.83%)	.003
Yes	600 (10.41%)	694 (12.01%)	588 (10.17%)	
AMI				
No	5216 (90.49%)	5400 (93.46%)	5570 (96.38%)	<.001
Yes	548 (9.51%)	378 (6.54%)	209 (3.62%)	
CHF				
No	5047 (87.56%)	4878 (84.42%)	5021 (86.88%)	<.001
Yes	717 (12.44%)	900 (15.58%)	758 (13.12%)	
Cirrhosis				
No	5726 (99.34%)	5686 (98.41%)	5565 (96.30%)	<.001
Yes	38 (0.66%)	92 (1.59%)	214 (3.70%)	
Pneumonia				
No	5032 (87.30%)	4668 (80.79%)	4367 (75.57%)	<.001
Yes	732 (12.70%)	1110 (19.21%)	1412 (24.43%)	
Diabetes				
No	4323 (75.00%)	4193 (72.57%)	4284 (74.13%)	.011
Yes	1441 (25.00%)	1585 (27.43%)	1495 (25.87%)	
Rhythm				
No	4423 (76.73%)	4378 (75.77%)	4432 (76.69%)	.386
Yes	1341 (23.27%)	1400 (24.23%)	1347 (23.31%)	
ICU 28-day mortality				
No	5430 (94.2%)	5310 (91.9%)	5074 (87.8%)	<.001
Yes	334 (5.8%)	468 (8.1%)	705 (12.2%)	

AST = aspartate transferase, ALT= alanine transferase, BMI = body mass index, COPD = chronic obstructive pulmonary disease, GCS = glasgow coma scale, RBC = red blood cell, WBC = white blood cell.

To verify the robustness of our outcomes, RAR levels were also evaluated as categorical variables, performing trend tests to assess consistency. A stratified analysis was executed using RAR-related variables as stratification factors to determine the relationship between RAR levels and 28-day mortality risk across diverse subgroups. Each subgroup variable was included in multivariable models to assess potential effect modification. All interaction analyses were exploratory and not specified a priori. Interaction tests were conducted using the log-likelihood ratio test.

Due to the continuous nature of RAR, potential nonlinear associations were also considered. Recognizing the limitations of binary logistic regression for nonlinear relationships, we utilized a generalized additive model with smooth curve fitting to explore these associations. In the event of a nonlinear relationship, a recursive algorithm was applied to identify the inflection point, followed by a 2-piecewise linear model to estimate OR and 95% confidence intervals for values on either side of the inflection point.

Kaplan–Meier survival curves were generated and the log-rank test was used to compare survival for different levels of RAR groups. Receiver operating characteristic curve analysis was applied to evaluate whether RAR possess the predictive value of 28-day mortality in sepsis.

All analyses were carried out using the statistical software packages R (https://www.R-project.org, The R Foundation) and EmpowerStats (https://www.empowerstats.com, X&Y Solutions, Inc, Boston, MA). A 2-sided *P*-value <.05 was considered statistically significant.

### 2.6. Ethics approval and consent to participate

Data was extracted from the eICU Collaborative Research Database (eICU-CRD) in accordance with the data usage agreement (our record ID: 40859994) by the PhysioNet review committee.^[21]^ The utilized database is released under the Health Insurance Portability and Accountability Act safe harbor provision. Informed consent to participate was obtained from all participants in the study. This was a retrospective analysis based on an anonymous database for researchers and obtained ethical approval from the Ethics Committee of the Affiliated Hospital of Guizhou Medical University.

## 3. Results

### 3.1. Patient selection procedure

Of the 200,859 patients in the eICU Collaborative Research Database (eICU-CRD) was used in this investigation. All patients were ≥60 years old, of which 56,245 records were excluded due to length of ICU stays <48 hours, and 21,362 records were excluded due to lack of RDW or albumin information. Ultimately, 17,321 older patients with sepsis were finally included in this study. The flow chart provides further information (Fig. 1).

### 3.2. Patient baseline characteristics

The participants in this study were then subdivided into the following 3 groups based on the RAR: <4.82 (n = 5764), 4.82 to 6.30 (n = 5778), and > 6.30 (n = 5779). Table 1 shows the baseline and clinical characteristics of the different RAR groups. Older patients with sepsis and a higher RAR tended to have a higher heart rate, WBC count and RDW. However, they had lower glasgow coma scale score, total protein, albumin, RBC count. They were also more likely to exhibit higher critical scores of Acute Physiology Score III and Apache IV score. The prevalence of cirrhosis, pneumonia and diabetes was also significantly increased in patients with a higher RAR group. More importantly, ICU 28-day mortality was significantly increased in higher RAR group.

### 3.3. Univariate and multivariate analyses of RAR

First, we performed a collinearity testing for variates presented in Table 1, the result was showed in Table S1, Supplemental Digital Content, https://links.lww.com/MD/Q597, VIF values less than 5 were chose. To investigate the relationship between the RAR and the outcomes of older patients with sepsis, we performed the various covariate correction procedures, extended multivariate models revealed significant associations between RAR and ICU 28-day mortality. The results were shown in Table 2. When analyzed as a continuous variable, RAR was remarkably associated with 28-day mortality.

**Table 2:** Relationship between RAR and ICU 28-day mortality in older patients with sepsis.

Outcome	Crude model		Model Ⅰ		Model Ⅱ	
	OR (95% CI)	*P*-value	OR (95% CI)	*P*-value	OR (95% CI)	*P*-value
RAR	1.15 (1.13–1.18)	<.001	1.16 (1.13–1.18)	<0.001	1.07 (1.04–1.09)	<.001
RAR (Tertile)						
<4.82	Ref.		Ref.		Ref.	
4.82–6.30	1.43 (1.24–1.66)	<.001	1.42 (1.23–1.65)	<0.001	1.11 (0.94–1.32)	.200
>6.30	2.26 (1.97–2.59)	<.001	2.25 (1.97–2.58)	<0.001	1.36 (1.16–1.59)	<.001
*P* for trend	<.001	–	<.001	–	<.001	–

CI = confidence interval, OR = odds ratio.

Model Ⅰadjusted for age and sex.

Model Ⅱadjusted for all covariates presented in Table 1.

In order to perform sensitivity analysis, we transformed RAR into categorical variables based on quartiles and calculated p-values for trend tests. We found consistent results when comparing RAR as a continuous or categorical variable. In the crude model, the OR (95% CI) for tertile 2 and tertile 3 were 1.43 (1.24–1.66) and 2.26 (1.97–2.59), respectively, compared with the reference group tertile 1 (*P* < .001). This association remained statistically significant even after adjusting for sex, age (Model Ⅰ). In model Ⅱ (adjusted for all covariates presented in Table 1), the adjusted OR (95% CI) was 1.11 (0.94–1.32) and 1.36 (1.16–1.59) for tertile 2 and tertile 3, respectively, compared to the reference group tertile 1 (*P* < .001). Higher RAR group were associated with higher risk of mortality.

### 3.4. Identification of nonlinear relationship

**Figure 2.**
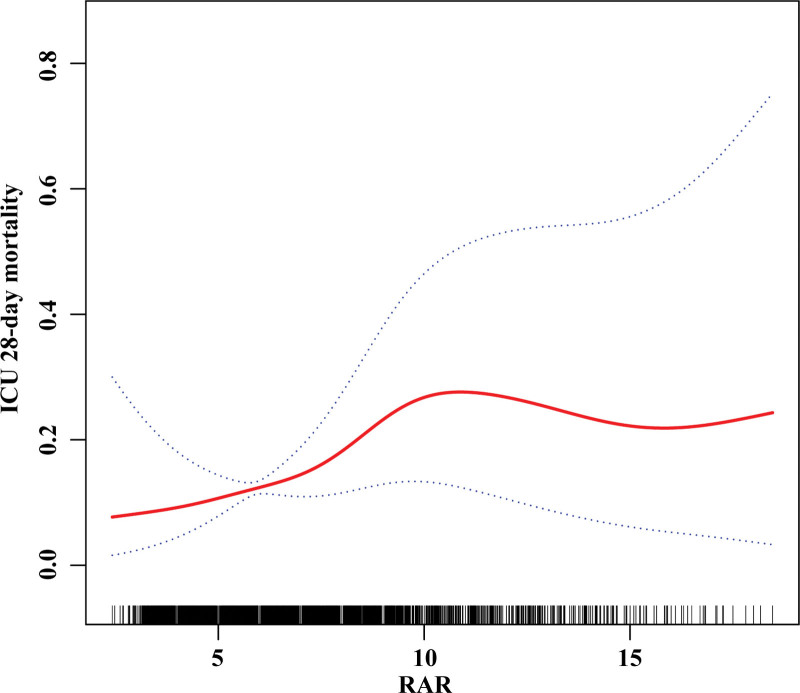
Associations between the RAR and 28-day mortality in older patients with sepsis. A threshold, nonlinear association between the RAR and 28-day mortality was found in a GAM. Solid rad line represents the smooth curve fit between variables. Blue bands represent the 95% of confidence interval from the fit. Adjusted for all covariates presented in Table 1. GAM = generalized additive model, RAR = red blood cell distribution width to albumin ratio.

To evaluate the linear association between RAR and mortality in older patients with sepsis, we performed a smooth curve fitting by generalized summation models. After adjusting for confounding variables presented in Table 1, a nonlinear association between RAR and 28-day mortality was observed (Fig. 2). We determined the inflection points using a 2-stage linear model and recursive techniques, which were found to be at 9.7. Our analyses indicated (Table 3) that when RAR levels were < 9.7, there was a 40% increase in the risk of 28-day mortality (OR: 1.4; 95% CI: 1.2 to 1.7, *P* < .001). However, we did not observe a significant association between RAR and 28-day mortality (OR: 1.0; 95% CI: 0.9 to 1.1, *P* = .897) when RAR levels were > 9.7. These findings suggest that the relationship between RAR and 28-day mortality risk may not follow a linear trend.

**Table 3:** Nonlinear association between RAR and ICU 28-day mortality in older patients with sepsis.

Models	OR, 95% CI, *P*-value
Fitting model by standard logistic regression model	1.1 (1.0–1.2) .212
Fitting model using 2-piecewise linear model Inflection point	–
Inflection point	9.7
<9.7	1.4 (1.2–1.7) <.001
>9.7	1.0 (0.9–1.1) .897
Log-likelihood ratio tests	<.001

Table 3 adjusted for all covariates presented in Table 1.

CI = confidence interval, OR = odds ratio.

### 3.5. Subgroup analysis

Subgroup analysis revealed the relationship between the RAR and 28-day mortality in older patients with sepsis with different comorbidities, including detailed p-values for each interaction (Table 4). Significant interactions were observed only for chronic obstructive pulmonary disease and RAR on 28-day mortality (*P* for interaction = 0.009). For chronic obstructive pulmonary disease patients, 28-day mortality was higher with increasing RAR. The OR (95%CI) for tertile 2 and tertile 3 were 1.18 (0.73–1.91) and 2.93 (1.91–4.51), respectively, compared with tertile 1 (*P* <.01). No significant interactions were observed in other subgroups (*P* for interaction >.05).

**Table 4:** Subgroup analysis of the associations between RAR and ICU 28-day mortality in older patients with sepsis.

Characteristic	No. of participants	RAR			*P*	*P* for interaction
		Tertile 1, <4.82	Tertile 2, 4.82–6.30	Tertile 3, >6.30		
Sex						
Male	8158	1.0	1.47 (1.18–1.84) 0.0006	2.50 (2.04–3.07)	<.001	.462
Female	9162	1.0	1.41 (1.17–1.72)	2.08 (1.73–2.50)	<.001	
Diabetes						
Yes	4521	1.0	1.38 (1.01–1.87)	2.41 (1.81–3.20)	<.001	.415
No	12,800	1.0	1.46 (1.24–1.72)	2.22 (1.90–2.59)	<.001	
CHF						
Yes	2375	1.0	1.34 (0.94–1.93)	1.98 (1.39–2.83)	<.001	.578
No	14,946	1.0	1.44 (1.23–1.69)	2.31 (1.99–2.67)	<.001	
AMI						
Yes	1135	1.0	1.83 (1.19–2.81)	2.05 (1.25–3.35)	<.001	.568
No	16,186	1.0	1.42 (1.21–1.66)	2.33 (2.02–2.69)	<.001	
Cirrhosis						
Yes	344	1.0	0.53 (0.11–2.49)	1.68 (0.48–5.86)	<.001	.099
No	16,977	1.0	1.45 (1.25–1.68)	2.26 (1.97–2.59)	<.001	
Pneumonia						
Yes	3254	1.0	1.30 (0.95–1.76)	1.94 (1.46–2.58)	<.001	.874
No	14,067	1.0	1.40 (1.18–1.65)	2.16 (1.84–2.52)	<.001	
Rhythm						
Yes	4088	1.0	1.45 (1.13–1.86)	1.97 (1.54–2.50)	<.001	.322
No	13,233	1.0	1.42 (1.18–1.70)	2.41 (2.05–2.85)	<.001	
COPD						
Yes	1882	1.0	1.18 (0.73–1.91)	2.93 (1.91–4.51)	<.001	.009
No	15,439	1.0	1.47 (1.26–1.71)	2.19 (1.90–2.53)	<.001	

AMI = acute myocardial infarction, CHF = congestive heart failure, COPD = chronic obstructive pulmonary disease, RAR = red blood cell distribution width to albumin ratio.

### 3.6. Predictive value of RAR

**Figure 3.**
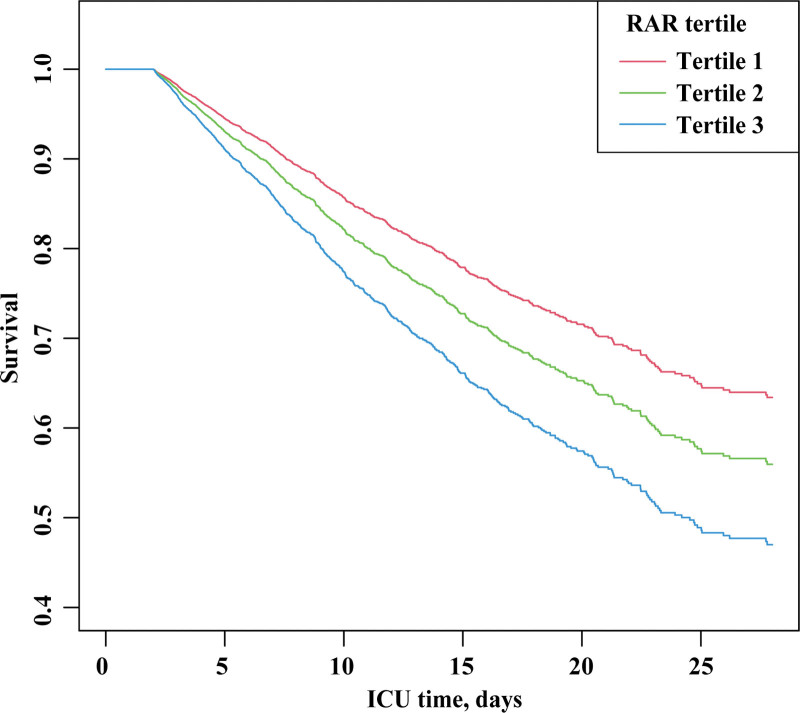
Kaplan–Meier curve of 28-day mortality for older patients with sepsis. (tertile 1: 5764 patients, tertile 2: 5778 patients, tertile 3: 5779 patients).

To assess cumulative survival at different levels of RAR, we generated 28-day survival curves for older patients with sepsis by stratifying according to the RAR tertiles. Kaplan–Meier analysis showed that patients in the low RAR group (tertile 1) had a significantly higher 28-day survival (*P* <.001, Fig. 3).

## 4. Discussion

This study investigated the potential association between the RAR and 28-day mortality in older patients with sepsis. Our findings suggest that a higher RAR is significantly associated with an increased risk of mortality in this patient population. These results highlighted the prognostic utility of RDW and albumin levels in various clinical contexts, including critical illnesses such as sepsis.

The increased RAR in non-survivors can be attributed to several pathophysiological mechanisms. Firstly, an elevated RDW may reflect underlying inflammatory processes and oxidative stress, which are prevalent in sepsis and contribute to poor outcomes.^[22]^ Secondly, low albumin levels, indicative of hypoalbuminemia, could denote malnutrition, chronic illness, or heightened vascular permeability, conditions that exacerbate sepsis severity and compromise patient survival.^[23,24]^

The growing body of literature suggesting that combining multiple biomarkers into a ratio like RAR can enhance prognostic accuracy. The global aging population faces significant challenges from frailty-a state of diminished strength and resilience that heightens vulnerability to disease, hospitalization, and mortality, driven by physiological decline and systemic inflammation.^[25]^ By integrating RDW and albumin levels, RAR captures both systemic inflammation and nutritional status, providing a more comprehensive overview of the patient’s condition. This dual marker approach may offer clinicians a simple and effective tool for risk stratification, allowing for more tailored and timely interventions in older sepsis patients. Moreover, previous research has increasingly highlighted the prognostic value of composite ratios that combine indicators of erythropoietic dysfunction, such as RDW, with markers of inflammation, immune response, or nutritional status, including albumin and lymphocyte counts, in septic populations.^[20,26,27]^ Studies utilizing RDW-to-albumin and RDW-to-lymphocyte ratios have reported enhanced risk stratification for adverse outcomes in sepsis and related critical illnesses. These findings collectively support the notion that integrating distinct pathophysiological markers – rather than relying on individual indices – can provide a more nuanced assessment of mortality risk among septic patients by capturing the combined effects of inflammation, immune dysregulation, and underlying nutritional or metabolic derangements.

However, this study has several limitations. Although hypoalbuminemia is a predictor of sepsis severity and mortality, it is also a well-established marker of chronic illness, malnutrition, and organ dysfunction. Low albumin may not solely act as a modifiable risk factor but also as a reflection of advanced disease or terminal physiological decline. In older patients, hypoalbuminemia could reflect accumulated comorbidities or late-stage disease, rather than an acute, modifiable process amenable to intervention. Moreover, as a retrospective cohort study, it is subject to potential biases related to data collection and inherent confounding variables. Furthermore, our findings may not be generalizable to all sepsis patients, as the study specifically focused on older adults. Prospective studies with larger sample sizes are needed to validate our findings and further explore the biological mechanisms underlying the association between RAR and mortality in sepsis. In addition, recent advancements in machine learning, such as the multicenter protocol demonstrate the feasibility of integrating imaging and functional biomarkers for frailty assessment.^[28]^ The methodology underscores the value of multimodal data in refining predictive accuracy-a direction that could further enhance the robustness of our research in future iterations.

## 5. Conclusions

In conclusion, our study demonstrates that a higher RAR is an independent predictor of 28-day mortality in older patients with sepsis. This suggests that RAR could be utilized as a convenient and effective prognostic marker in clinical settings to identify high-risk patients and guide management decisions. Further research is necessary to confirm these results and to explore potential interventions that could modify RAR and improve outcomes.

## Author contributions

**Conceptualization:** Kai Hu.

**Data curation:** Kai Hu.

**Funding acquisition:** Husun Qian.

**Investigation:** Kai Hu.

**Methodology:** Kai Hu.

**Project administration:** Kai Hu.

**Resources:** Kai Hu.

**Supervision:** Husun Qian.

**Visualization:** Husun Qian.

**Writing – original draft:** Kai Hu.

**Writing – review & editing:** Husun Qian.

## Supplementary Material


