# Supplementary material for: Association between red cell distribution width to albumin ratio and 28‑day mortality in older patients with sepsis: A retrospective cohort study
Source: Medicine (Baltimore). 2025 Nov 7;104(45):e45835. doi: 10.1097/MD.0000000000045835 (PMC12599801; doi:10.1097/MD.0000000000045835)
Supplement: Supplementary file 1 [file medi-104-e45835-s001.docx]

Supplementary Material

| Characteristic | VIF |
| --- | --- |
| Age (years) | 1.1 |
| Gender | 1.0 |
| BMI | 1.1 |
| WBC | 1.1 |
| Temperature | 1.2 |
| Heart rate | 1.1 |
| Acute Physiology Score III | 1.4 |
| Apache IV score | 1.6 |
| GCS score | 1.5 |
| AST | 1.3 |
| ALT | 2.0 |
| Total protein | 1.5 |
| Albumin | 1.4 |
| Lactate | 1.1 |
| RBC | 1.6 |
| RDW | 4.2 |
| COPD | 1.5 |
| AMI | 1.0 |
| CHF | 1.1 |
| Cirrhosis | 1.4 |
| Pneumonia | 11 |
| Diabetes | 1.1 |
| Rhythm | 1.1 |

Table S1. Collinearity testing for variates.
